# Grapefruit Debittering by Simultaneous Naringin Hydrolysis and Limonin Adsorption Using Naringinase Immobilized in Agarose Supports

**DOI:** 10.3390/molecules27092867

**Published:** 2022-04-30

**Authors:** Mariela Muñoz, Jessica Holtheuer, Lorena Wilson, Paulina Urrutia

**Affiliations:** School of Biochemistry Engineering, Pontificia Universidad Católica de Valparaíso, Av. Brasil 2950, Valparaíso 2362803, Chile; mariela.mubu@gmail.com (M.M.); jessica_holth@hotmail.com (J.H.); lorena.wilson@pucv.cl (L.W.)

**Keywords:** immobilization, naringinase, limonin, naringin, debittering, grapefruit juice

## Abstract

Naringin and limonin are the two main bitter compounds of citrus products such as grapefruit juice. The aim of this investigation was to evaluate the reduction in both bitter components simultaneously using a combined biochemical and physical approach. The proposed strategy was based on the use of heterofunctional supports with glyoxyl groups that allow for the covalent immobilization of naringinase, which hydrolyses naringin and alkyl groups that allow for the adsorption of limonin. The supports were butyl-glyoxyl agarose (BGA) and octyl-glyoxyl agarose (OGA), which were characterized in terms of aldehyde group quantification and FTIR analysis. The optimal pH and temperature of free and immobilized enzymes were assessed. The maximum enzyme loading capacity of supports was analyzed. Debittering of grapefruit juice was evaluated using soluble enzyme, enzyme-free supports, and immobilized catalysts. Enzyme immobilized in BGA reduced naringin and limonin concentrations by 54 and 100%, respectively, while the use of catalyst immobilized in OGA allowed a reduction of 74 and 76%, respectively, obtaining a final concentration of both bitter components under their detection threshold. The use of OGA biocatalyst presented better results than when soluble enzyme or enzyme-free support was utilized. Biocatalyst was successfully applied in juice debittering in five repeated batches.

## 1. Introduction

Taste is one of the sensory quality attributes that, together with color and flavor, determines food acceptance. Bitterness in citrus juices reduces the quality and commercial value of the product, which has been a long-standing problem [1,2]. There are two types of bitter compounds in citrus juices, limonoids and flavonoids. Limonoids are highly oxygenated triterpenes, classed as tetranorterpenoids. They have moderate polarity and are present in neutral (noncarboxylated/aglycon) as well as acidic (carboxylated/glucoside) forms. The former is insoluble and bitter, while the latter is soluble and tasteless [3]. The main limonoid present in citrus juices is limonin, which is responsible for “delayed” bitterness that develops after juice extraction [2,4]. Intact fruit barely contains limonin; however, its non-bitter precursor, limonate-A-ring lactone (LARL), is present in cell cytoplasm in membranous sacs. When these sacs are ruptured during juice processing, the acidic pH of the juice gradually catalyzes the closure of the LARL ring, forming limonin [2,5]. The threshold for limonin in orange juice is affected by pH and is 6.5 ppm at pH 3.8 [6]. Meanwhile, flavonoids make up a large group of very different compounds that share the common feature of phenol moieties. They are mainly present in citrus fruits as glycosyl derivatives. The forms lacking sugar moieties (aglycone) occur less frequently owing to their lipophilic nature and low solubility in water [7]. Hesperetin and naringenin are the most common flavanones in fruits, and they are usually conjugated to glucose–rhamnose disaccharide at the 7-position, typically rutinose or neohesperidose. Flavanone aglycones and rutinosides are tasteless, whereas flavanone neohesperidose conjugates, such as naringin in grapefruit (*Citrus paradasi*) and neohesperidin in bitter orange (*C. aurantium*), are intensely bitter [8]. Narinigin is found in the membranes and albedo of the fruits and is extracted into the juice, giving it an “immediate” bitterness when the levels exceed 20 ppm [9].

**Scheme 1:**
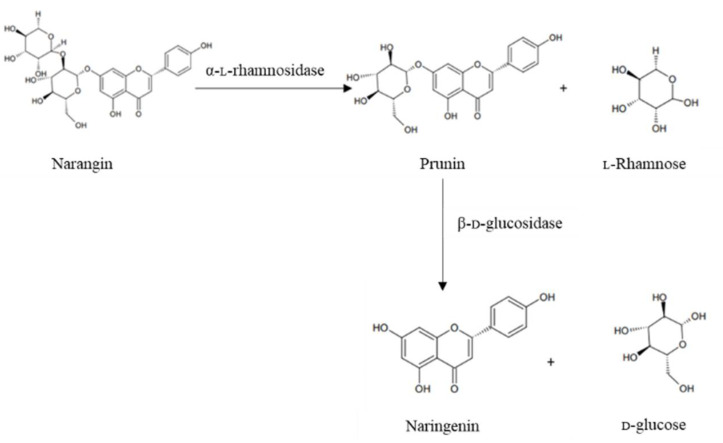
Hydrolysis of naringin into prunin, rhamnose, naringenin, and glucose by sequential reaction of α-rhamnosidase and β-glucosidase.

Bitterness due to flavonoids and limonoids poses a major problem for the citrus industry, and without proper debittering technology, the industry cannot flourish [10]. Due to the importance of reducing or removing bitterness in citrus juices below the threshold level for consumer acceptability, several physicochemical and biochemical strategies have been developed. Among the physicochemical approaches, the use of adsorptive and/or ion-exchange resins is preferred for the removal of bitter compounds based on their easy handling and the possibility of regeneration for long-term use. Several natural and synthetic hydrophobic and hydrophilic adsorbents have been tested. Neutral adsorbent resins have shown preferential adsorption of limonin over naringin, probably due to limonin’s greater hydrophobicity, with reported adsorption rates of up to 73% for naringin and 85−95% for limonin present in citrus juice [11,12,13,14,15]. In the case of ion-exchange resins, weakly basic anion exchange resins have been effectively used to reduce the concentration of bitter compounds and may also have an efficient function in adjusting the taste equilibrium of products since they also reduce the acidity of the juice [16,17]. However, these techniques involve the removal of not only the bitter compounds but also the nutrients/flavor/color from the juice [18,19,20,21]. Due to some drawbacks associated with physicochemical treatments, the enzymatic conversion of bitter compounds has been investigated as an alternative process. Even though in the case of limonin, the use of the enzyme limonoate dehydrogenase for the oxidation of LARL to 17-dehydrolimonoate, a non-bitter derivate, has been reported, its application at the acidic pH of fruit juices has been difficult due to its optimal alkaline pH [22]. For this reason, reducing juice bitterness using enzymes is mainly based on converting naringin by the enzyme naringinase. Naringinase (EC 3.2.1.40) is a hydrolytic enzyme containing both α-l-rhamnosidase and β-d-glucosidase, which are located on two separate polypeptides [23]. As can be observed in Scheme 1, first, α-l-rhamnosidase hydrolyzes naringin into rhamnose and prunin (4,5,7-trihydroxy flavanone-7-glucoside), then the prunin is hydrolyzed into glucose and naringenin (4,5,7-trihydroxy flavanone) by β-d-glucosidase activity [24]. Prunin is 33% less bitter than naringin, and its further hydrolysis reduces the bitter taste even more [2].

The debittering of citrus juices by immobilized naringinase has been reported [19]. Different supports and immobilization methods have been utilized, including entrapment in beads of natural polymers (alginate and k-carrageenan), removal of 70−95% of naringin present in different citrus juices [15,25,26,27], and entrapment in cellulose triacetate fibers, which removes up to 35% of naringin and 58% of limonin in grapefruit [28]. Covalent immobilization of naringinase has also been reported using different supports, including alginate beads functionalized with aldehyde groups [29,30], chitosan activated with glutaraldehyde [31], glutaraldehyde-coated wood chips [32], glutaraldehyde-coated hen egg whites [33], milled bovine horns, sheep wool and silk fibers functionalized with glutaraldehyde [34], two-dimensional zeolite derivatized with glutaraldehyde [35], silica functionalized with glutaraldehyde [36], and glutaraldehyde cross-linking on the surface of polyethylenimine/dopamine-coated hydrothermal carbon [37]. The use of covalently immobilized naringinase in the treatment of citrus juices has resulted in the removal of 68−76% of the original naringin [32,33]. The use of naringin solution as a substrate in conversions of 27% to >90% has been reported [35,36]. Despite the many attractive features of the enzymatic process, some reasons for its limited application at present are the cost and availability of commercial enzymes and the fact that the limonin content is not at all affected by naringinase treatment [38].

**Scheme 2:**
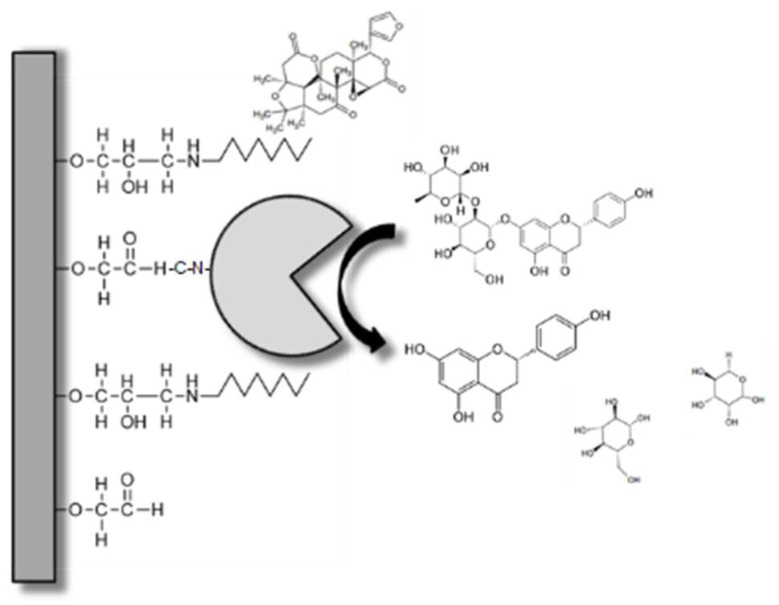
Scheme of the system proposed for the simultaneous hydrolysis of naringin and adsorption of limonin.

In this study, a single strategy combining physical and biochemical principles for debittering citrus juice by the simultaneous hydrolysis of naringin and adsorption of limonin was evaluated. Naringinase was covalently immobilized in the heterofunctional supports butyl-glyoxyl agarose (BGA) and octyl-glyoxyl agarose (OGA) through glyoxyl groups as carriers, while the alkyl chains of the supports were utilized for limonin adsorption (Scheme 2). The biocatalysts were characterized, and the debittering of grapefruit juice was evaluated using soluble enzymes, enzyme-free supports, and immobilized biocatalysts. Enzyme immobilized in OGA was selected and applied in juice debittering in repeated batch operation.

## 2. Results and Discussion

### 2.1. Characterization of Naringinase

The enzymatic preparation of Novozyme NS 33117 was characterized in terms of its protein content and activity using substrates *p*-nitrophenyl-β-*d*-glucoside (*p*NPG) and *p*-nitrophenyl-α-l-rhamnopyranoside (*p*NPR), and the results are shown in Table 1.

**Figure 1:**
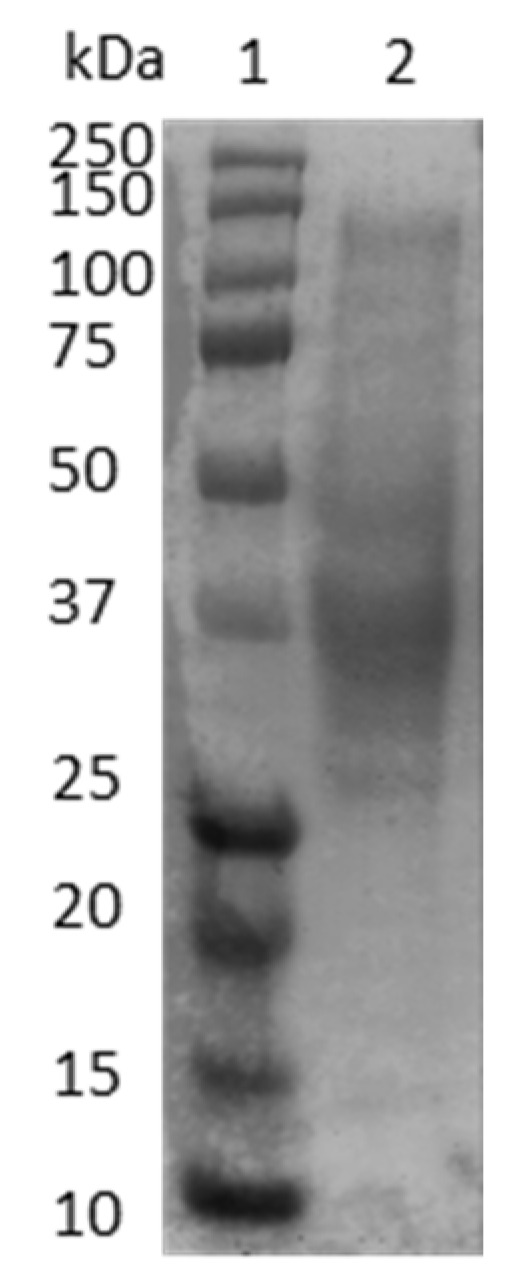
SDS-PAGE: Lane 1: low-molecular-weight standard (Precision Plus Protein, Bio-Rad); Lane 2: naringinase diluted 80 times in distilled water.

The molecular weight of the enzyme was analyzed by SDS-PAGE electrophoresis (Figure 1). It presented three main bands of 100−150, 50−37, and 37−25 kDa, values that are not in total accordance with those previously reported; however, it should be considered that the utilized naringinase was an enzymatic preparation from A. aculeatus and A. niger and the molecular weight of naringinase varied depending on its origin and fermentation conditions [23,38,39]. In the case of naringinase from A. aculeatus, Chen et al. (2013) reported that the enzyme had a molecular mass of 348 kDa and contained four subunits of 100, 95, 84, and 69 kDa, three of which corresponded to β-d-glucosidase subunits and one corresponded to an α-l-rhamnosidase subunit [40]. In the case of A. niger, Borka et al. (2010) reported that naringinase from A. niger van Tieghem MTCC 2425 had molecular weight bands of 10–20, 65, and 80 kDa [41], while Puri and Kalra (2005) found that naringinase from A. niger 1344 corresponded to a heterodimer of 168 kDa [42], and Zhu et al. (2017) reported just one molecular weight band of 23 kDa for both α-l-rhamnosidase and β-d-glucosidase using A. niger 11250 [43].

**Figure 2:**
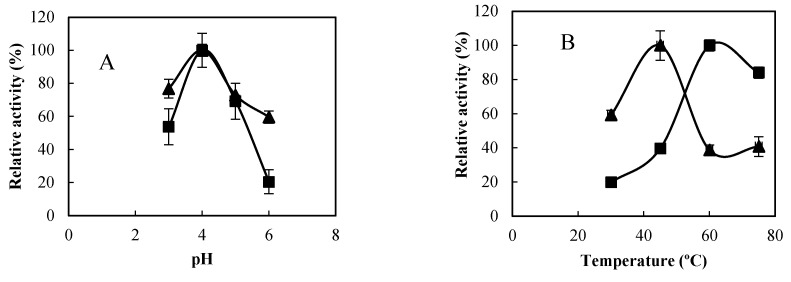
Effect of (**A**) pH and (**B**) temperature on the relative activity of free naringinase using, as substrate, ∎ *p*-nitrophenyl-β-D-Glucoside (*p*NPG) and ⯅ *p*-nitrophenyl-α-l-rhamnopyranoside (*p*NPR).

The impact of pH and temperature on the activity of naringinase was analyzed (Figure 2). The pH that results in the maximal *p*NPG and *p*NPR activities was found to be pH 4.0. This value is in accordance with the optimal pH previously reported for α-l-rhamnosidase and β-d-glucosidase from *A. aculeatus* [40]. In the case of naringinase from *A. niger*, optimal pH values of 4.0 [42] and 5.0 [43] have been reported. The optimal temperature varies according to the substrate utilized; it was found to be 45 *°*C for *p*NPG and 60 *°*C for *p*NPR. Even though the values differ, the higher optimal temperature for β-d-glucosidase is in accordance with that observed for naringinase from *A. aculeatus* [40]. In the case of naringinase from *A. niger*, optimal temperatures of 45 °C [44] and 50 °C [42] have been reported.

### 2.2. Immobilization of Naringinase in BGA and OGA

**Figure 3:**
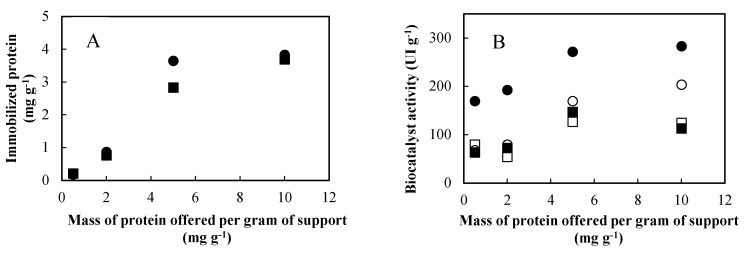
Maximum loading capacity of ● BGA and ∎ OGA in terms of (**A**) mass of protein immobilized and (**B**) biocatalyst activity. Open and closed symbols in (**B**) represent the activity of *p*NPG and *p*NPR, respectively.

The heterofunctional supports BGA and OGA were produced by oxidation of butyl and octyl agarose, respectively. Characterization of supports can be found in Supplementary Materials. The enzyme was immobilized in BGA and OGA, and the maximum enzyme loading capacity of the supports was determined (Figure 3). As can be observed, in both supports, the maximum amount of protein immobilized was approximately 4 mg g^−1^, without significant differences between them. This value is low in comparison with the 20−30 mg mL^−1^ reported for glyoxyl agarose 4 BCL [44], a support with the same concentration of agarose that is activated with a similar quantity of glyoxyl groups but without the alkyl groups. Considering that the immobilization through glyoxyl groups is multipoint immobilization [45], the low maximum protein immobilization may be associated with a low density of lysin residues in the naringinase surface or their homogenous distribution [44]. Additionally, it has been reported that the occurrence of steric hindrance in the enzyme–support reaction is a critical factor when this reaction is intended to be maximized [46]; therefore, it may be possible that the bigger spacer arm of hydrophobic functional groups could also affect the enzyme-support interaction required for immobilization. In terms of biocatalyst activity, the maximum values were obtained when using 5 mg of protein per g of support, and higher activity was expressed when BGA was utilized.

**Figure 4:**
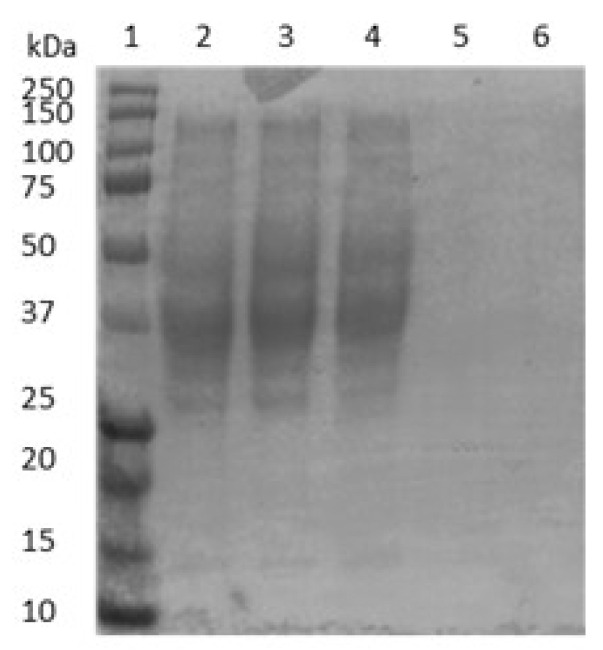
SDS-PAGE: Lane 1: low-molecular-weight standard (Precision Plus Protein, Bio-Rad); Lanes 2 and 3: supernatant at 24 h of immobilization in BGA and OGA, respectively; Lane 4: control sample (supernatant of BGA at t = 0 of immobilization); Lanes 5 and 6: catalyst immobilized in BGA and OGA, respectively.

To assess the covalent immobilization process, the supernatant obtained after 24 h of immobilization, the control sample (supernatant at t = 0 of immobilization), and the immobilized biocatalysts were analyzed by SDS-PAGE electrophoresis (Figure 4). As can be observed, no band appeared when immobilized enzymes were analyzed, indicating that covalent bonds between enzyme and support were produced.

**Figure 5:**
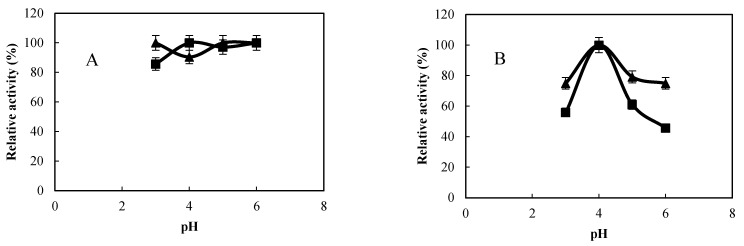

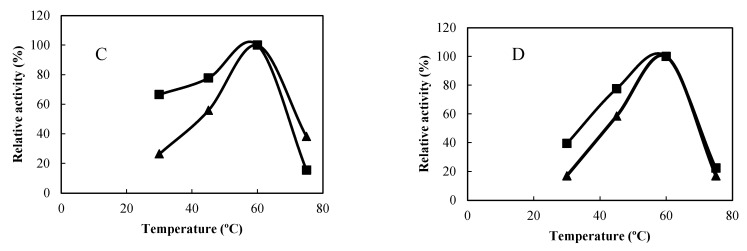
Effect of (**A**,**B**) pH and (**C**,**D**) temperature on the initial reaction rate of enzymes immobilized in BGA (**A**,**C**) and OGA (**B**,**D**) using ∎ *p*NPG and ⯅ *p*NPR.

The impact of immobilization on the optimal pH and temperature of enzymes was analyzed (Figure 5). As can be observed, immobilization in BGA resulted in a less pH-dependent activity profile of both α-l-rhamnosidase and β-d-glucosidase activity, while for OGA, this effect was detected only for α-l-rhamnosidase activity. This effect has been previously reported, and it might be attributed to the restriction of conformational changes due to pH [47,48]. Regarding temperature, immobilization in BGA and OGA increased the optimal temperature for α-l-rhamnosidase activity, while no changes were observed for β-d-glucosidase. The increased optimal temperature of α-l-rhamnosidase may be explained by an increase in thermal stability due to immobilization.

### 2.3. Debittering Grapefruit Juice

**Figure 6:**
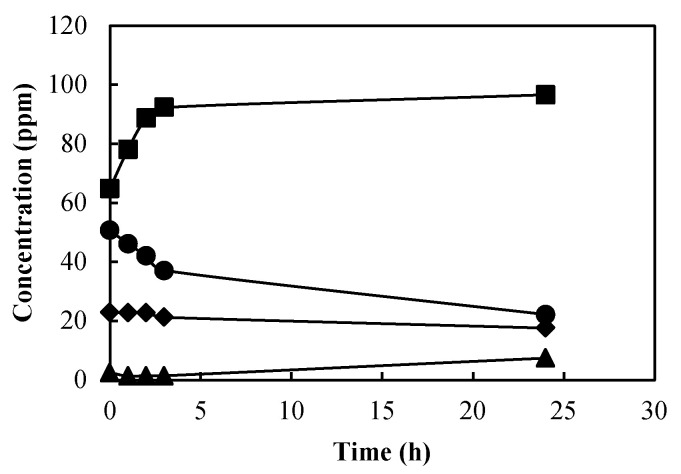
Kinetics of grapefruit juice debittering catalyzed by free enzyme: ⯁ Limonin; ● Naringin; ∎ Prunin; ⯅ Naringenin.

First, the commercial enzymatic preparation (free enzyme) was used for grapefruit juice debittering. Figure 6 shows the kinetics of naringin, prunin, naringenin, and limonin concentrations. Naringin concentration decreased over time with a subsequent increase in prunin concentration, reflecting the α-l-rhamnosidase activity. After 24 h of reaction, there was a 56% reduction in the initial concentration of naringin. Naringenin concentration increased after 24 h of reaction, indicating lower β-d-glucosidase activity. Limonin was not affected by the action of the enzyme, as expected, and a 23% reduction after 24 h was observed in a control sample (data not shown), which may be explained by the decomposition of the molecule.

**Figure 7:**
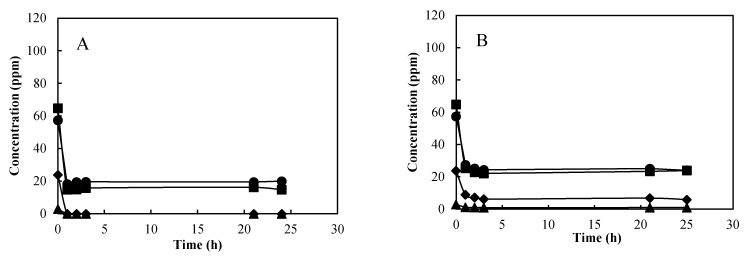
Kinetics of grapefruit juice debittering using enzyme-free supports: (**A**) BGA; (**B**) OGA. ⯁ Limonin; ● Naringin; ∎ Prunin; ⯅ Naringenin.

Before applying immobilized biocatalysts, the adsorption capacity of the supports was evaluated (Figure 7). Both enzyme-free supports allowed for the adsorption of all bitter compounds; however, the best performance was observed with BGA, which showed 100% removal of limonin and 65% of naringin after 24 h. In both cases, the adsorption of bitter molecules varied according to their hydrophobicity, and the reduction in initial concentration followed the order: limonin > naringenin > prunin > naringin. The preferential adsorption of limonin over naringin has also been observed with the use of neutral resin, as in the case of XAD-4 and XAD-7 [13].

**Figure 8:**
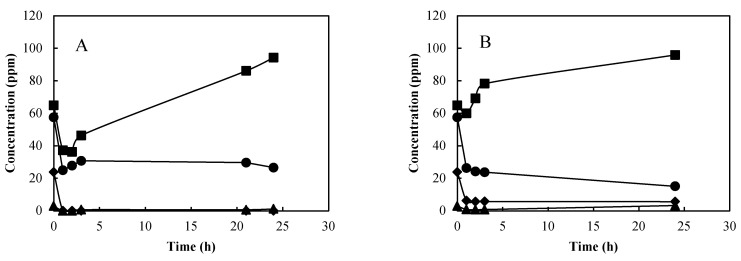
Kinetics of grapefruit juice debittering using enzyme immobilized in (**A**) BGA and (**B**) OGA. ⯁ Limonin; ● Naringin; ∎ Prunin; ⯅ Naringenin.

Finally, immobilized biocatalysts were used for grapefruit juice debittering. Figure 8 shows the kinetics of the reactions using enzyme immobilized in BGA and OGA. In both cases, in the first hours of reaction, there was a significant decrease in the concentration of all molecules, as was observed when enzyme-free supports were utilized. However, after this period, the concentration of prunin increased, reflecting the action of the enzyme, specifically α-l-rhamnosidase. Naringenin concentration also increased after 24 h of reaction, but at a much lower concentration, indicating lower activity of β-d-glucosidase, as was observed previously with the free enzyme. After 24 h of reaction, BGA and OGA catalysts resulted in 100 and 76% removal of limonin, respectively, while for naringin, the initial concentration was reduced by 54 and 75%, respectively. Limonin concentration after the debittering process was below the detecting threshold [6], independent of the support utilized for enzyme immobilization. In the case of naringin, only biocatalyst immobilized in OGA allowed for a final concentration below its detection threshold [9]. The use of immobilized catalysts resulted in a hybrid approach for juice debittering, with enzyme immobilized in OGA obtaining better results than soluble enzyme alone or enzyme-free support. These results were not observed in other investigations when naringinase was immobilized in a carrier with a hydrophilic surface [47]. To the best of our knowledge, only Tsen and Yu (1991) carried out simultaneous removal of limonin and naringin by naringinase immobilized in a hydrophobic support, with cellulose triacetate fibers used to entrap the enzyme; however, only 31−35% of naringin and 52−58% of limonin were removed from grapefruit juice [28]. These results show that tailor-made functionalization of the support is an attractive strategy to address the problem of bitterness in citrus juice.

**Figure 9:**
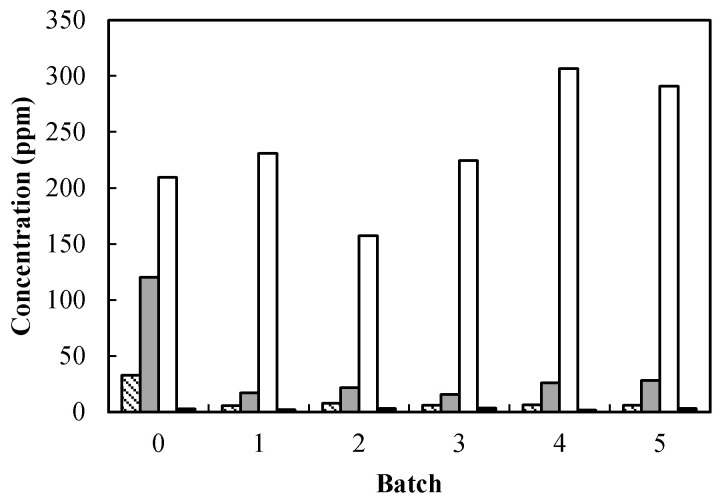
Grapefruit juice debittering in repeated batch operations using naringinase immobilized in OGA. Concentration of bitter compounds after 24 h of reaction. 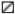 Limonin; 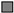 Naringin; □ Prunin; ∎ Naringenin.

The enzyme immobilized in OGA was used for juice debittering in repeated batch mode (Figure 9). The initial concentration of bitter compounds was higher than before, and this may be associated with the use of a natural substrate where the composition may be affected by season and plant growth phase, among other factors [49,50]. As can be observed, among five sequential batches, there was a reduction in the initial concentrations of limonin and naringin, reaching values close to their corresponding threshold. Except for batch 2, prunin concentration increased after the reaction, showing the α-l-rhamnosidase activity of the biocatalyst.

**Figure 10:**
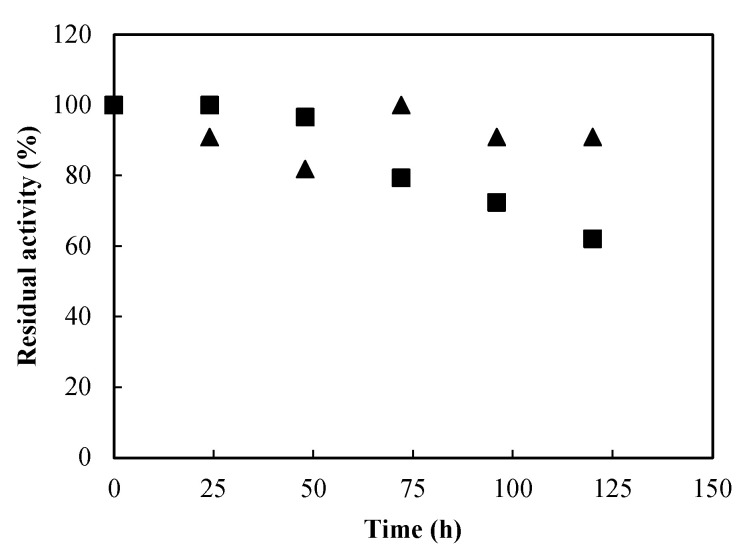
Stability under reactive conditions of naringinase immobilized in OGA. Activity of α-l-rhamnosidase and β-d-glucosidase using as substrate ∎ *p*NPG; ⯅ *p*NPR.

In order to corroborate the stability of the biocatalyst across repeated batch operations, the α-l-rhamnosidase and β-d-glucosidase activity was evaluated after each period of 24 h, using the synthetic substrates *p*NPR and *p*NPG. As can be observed in Figure 10, β-d-glucosidase had lower stability than α-l-rhamnosidase, and after 120 h of reaction, α-l-rhamnosidase maintained 90% of its initial activity.

## 3. Materials and Methods

### 3.1. Materials

Naringinase from *Aspergillus aculeatus*/*Aspergillus niger* (Novozyme NS 33117) was kindly donated by Novozyme. Naringin (≥95% HPLC), limonin (>90% HPLC), *p*-nitrophenyl-α-l-rhamnopyranoside (*p*NPR), and *p*-nitrophenyl-β-d-glucoside (*p*NPG) were purchased from Sigma-Aldrich, Santiago, Chile. Acetonitrile, octyl-sepharose 4 Fast Flow, butyl-sepharose 4 Fast Flow, and sodium metaperiodate were purchased from Merck, Santiago, Chile. All other chemicals were of analytical grade. Grapefruits were purchased from the local market.

### 3.2. Analytical Methods

The enzyme activity of naringinase was defined as the amount of enzyme needed to produce 1 µmol of *p*-nitrophenol (*p*NP) per minute from a solution at pH 4.0 and 45 °C containing 5 mM *p*NPR or 10 10 mM *p*NPG. In this study, 25 μL of the enzyme solution or suspension was added to 1 mL of sodium citrate buffer 25 mM (45 °C, pH 4.0) containing 5 mM *p*NPR or 10 mM *p*NPG. Every 2 min, 50 µL aliquot was withdrawn, and 3 mL of NaOH (0.5 M) was added to stop the reaction, and the absorbance was measured at 405 nm using a Jenway 6715 UV/Vis spectrophotometer. The extinction coefficient of *p*NP under assay conditions was 6.7484 M^−1^ cm^−1^. Protein concentration was measured by the Bradford method, using bovine serum albumin as standard.

The concentration of substrates and products of juice debittering were assessed by HPLC analysis (JASCO-DAD HPLC). Naringin, prunin, naringenin, and limonin were detected under UV light (210−280 nm). Separation was performed on a C-18 (15 cm × 0.4 cm) analytical column at 35 °C, and the flow rate of the mobile phase was 0.5 mL min^−1^. The injection volume was 20 µL, and the mobile phase was a gradient of acetonitrile in water with the following gradient program: 5:95 (*v*/*v*) for 4 min, then to 40:60 (*v*/*v*) in 10 min, then held at 40:60 (*v*/*v*) for 2 min, then to 70:30 (*v*/*v*) in 8 min, then to 5:95 (*v*/*v*) in 4 min, and then held at 5:95 (*v*/*v*) for 4 min.

The characterization of naringinase molecular weight and its possible release from the supports after enzyme immobilization was assessed by SDS–polyacrylamide gel electrophoresis using Mini-PROTEAN Tetra-Cell (Bio-Rad) and 12% Mini-PROTEAN^®^TGX^TM^ Precast Protein Gels (Bio-Rad). A 15 µL sample of the enzymatic solution was mixed with 15 µL of rupture buffer (β-mercaptoethanol, Merck) and boiled for 5 min, and a 20 µL aliquot of the supernatant was used in the experiments. In the case of immobilized biocatalysts, 10 mg of immobilized enzyme was suspended in 110 µL of rupture buffer (β-mercaptoethanol, Merck) and boiled for 5 min, and a 20 µL aliquot of the supernatant was used in the experiments. Gels were stained with Coomassie brilliant blue. Precision Plus Protein^TM^ Standards Kaleidoscope molecular weight marker (10–250 kDa) (Bio-Rad) was used.

### 3.3. Preparation of Supports

The supports were prepared by simple periodate oxidation of the commercial butyl agarose (BA) and octyl agarose (OA). The preparation of both heterofunctional supports was as follows: 10 g of commercial product was washed and filtered 5 times with distilled water, then suspended in 50 mL of sodium periodate (10 mM) and gently stirred for 2 h at 25 °C. Finally, the supports were filtered and washed with distilled water.

### 3.4. Immobilization of Naringinase in BGA and OGA

The immobilization of enzyme in BGA and OGA was performed as follows: 1 g of support was gently mixed with 10 mL of enzyme solution (25% glycerol, bicarbonate buffer 100 mM, pH 10.05) for 24 h at 4 °C using a roller mixer. In order to reduce reversible Schiff’s base to secondary amino bonds and unreacted aldehyde groups to fully inert hydroxyl groups, solid sodium borohydride was added to enzyme–support suspensions to a concentration of 1 mg mL^−1^, with gentle stirring for 30 min. After that, the immobilized naringinase was separated using a paper filter and washed with distilled water.

### 3.5. Effect of pH and Temperature on Catalytic Activity

The optimal pH value for free and immobilized naringinase was determined at 45 °C, adding 25 µL of catalyst solution (DF = 10) or 100 mg of immobilized biocatalysts to 1 mL of *p*NPR (5 mM) or *p*NPG (10 mM) solution prepared in sodium citrate buffer 25 mM. The pH varied from 3.0 to 6.0. Every 30 min, 50 µL was withdrawn and added to 3 mL of NaOH (0.5 M) and analyzed by the Jenway UV–VIS spectrometer at 405 nm. In the case of optimal temperature, it was determined to use the same procedure but at pH 4.0, and the temperature varied from 30 to 75 °C.

### 3.6. Adsorption of the Bitter Compound in Fresh Juice

To evaluate the adsorption of bitter compounds on supports, a support: juice ratio of 1 g: 1.25 mL was utilized. Freshly squeezed grapefruit juice was previously filtered (Whatman filter paper), and the mixture was covered with light and incubated in a shaker at 150 rpm and 30 °C. Samples of 200 µL of the supernatant were withdrawn periodically, centrifuged, and filtered with a 0.45 µm pore diameter. After that, the samples were analyzed to evaluate limonin, naringin, prunin, and narangenine concentrations by HPLC.

### 3.7. Debittering of Grapefruit Juice by Soluble and Immobilized Enzyme

To evaluate the debittering of grapefruit juice by soluble and immobilized enzyme, a catalyst: juice ratio of 1 mL:24 mL and 1 g:1.25 mL, respectively, was utilized. Freshly squeezed grapefruit juice was previously filtered (Whatman filter paper), and the mixture was covered with light and incubated in a shaker at 150 rpm and 30 °C. Samples of 200 µL of the supernatant were withdrawn periodically, centrifuged, and filtered through filter paper with a 0.45 µm pore diameter. After that, the samples were analyzed by HPLC to evaluate limonin, naringin, prunin, and naringenin concentrations.

To carry out the debittering of grapefruit juice under repeated batch operation using the OGA catalyst, after 24 h of reactions, the suspension was filtered, and the catalyst was washed with distilled water. The catalyst was recovered and used for another batch of reaction, maintaining the initial catalyst: juice ratio. The stability of naringinase immobilized in OGA under reactive conditions was assessed by measuring the *p*NPR and *p*NPG activity of the catalyst after each reaction batch.

## Figures and Tables

**Table 1:** Characterization of naringinase.

Protein Concentration (mg mL^−1^)	*p*NPR Activity (µmol min^−1^ mg_prot_^−1^)	*p*NPG Activity (µmol min^−1^ mg_prot_^−1^)
5.2 ± 0.8	1567.9 ± 251.9	5434.4 ± 75.6

## Supplementary Materials

The following supporting information can be downloaded at: https://www.mdpi.com/article/10.3390/molecules27092867/s1, Figure S1. FTIR spectra of commercial supports butyl agarose (BA) and octyl agarose (OA) and heterofunctional supports butyl-glyoxyl agarose (BGA) and octyl-glyoxyl agarose (OGA). References [51,52,53] are cited in Supplementary Materials.

## Sample Availability

Not available.
