# Supplementary material for: Grapefruit Debittering by Simultaneous Naringin Hydrolysis and Limonin Adsorption Using Naringinase Immobilized in Agarose Supports
Source: Molecules. 2022 Apr 30;27(9):2867. doi: 10.3390/molecules27092867 (PMC9103998; doi:10.3390/molecules27092867)
Supplement: Supplementary file 1 [file molecules-27-02867-s001.zip › molecules-1674946-supplementary.pdf]

molecules

MDPI

Supplementary material

Grapefruit debittering by simultaneous naringin hydrolysis and limonin adsorption using naringinase immobilized in agarose supports

Mariela Muñoz, Jessica Holtheuer, Lorena Wilson, and Paulina Urrutia

Characterization of supports

Butyl agarose (BA) and octyl agarose (OA) correspond to 4% cross-linked agarose with the aliphatic ligand bonded to the matrix by an ether linkage. Diol groups of commercial supports are produced by opening the epoxy groups during agarose cross-linking and modifying epoxy agarose to produce BA or OA, and it has been reported that the number of diol groups is in the range of 30 to 60 μmol per gram of support [51]. Aldehyde groups are introduced by the oxidation of commercial support diols to obtain the heterofuncional supports BGA and OGA. Aldehydes were quantified by back-titration of $NaIO_4$, with values of 44 and 39 μmol per gram of wet BGA and OGA, respectively, which are in the range previously reported [51]. The moisture of supports was 94%, with no significant difference between carriers. The chemical composition of supports was analyzed by FTIR (Nicolet iS 10), and the characteristic bands of the commercial and heterofunctional supports are shown in Figure S1. As can be observed, the biopolymer structure is reflected in the bands at 1380 cm^{-1} for C-C stretch of aromatic ring [52]; 1155 and 1070 cm^{-1} for C-O-C and glycosidic linkage; and 775, 885, and 930 cm^{-1} for 3,6-anhydro-β-galactose skeletal bending in agarose [53]. No alteration of these bands was observed after oxidation of supports BA and OA. In the case of BGA, a band at 1750 cm^{-1} was observed, which was attributed to the C=O functional group of the aldehyde groups.

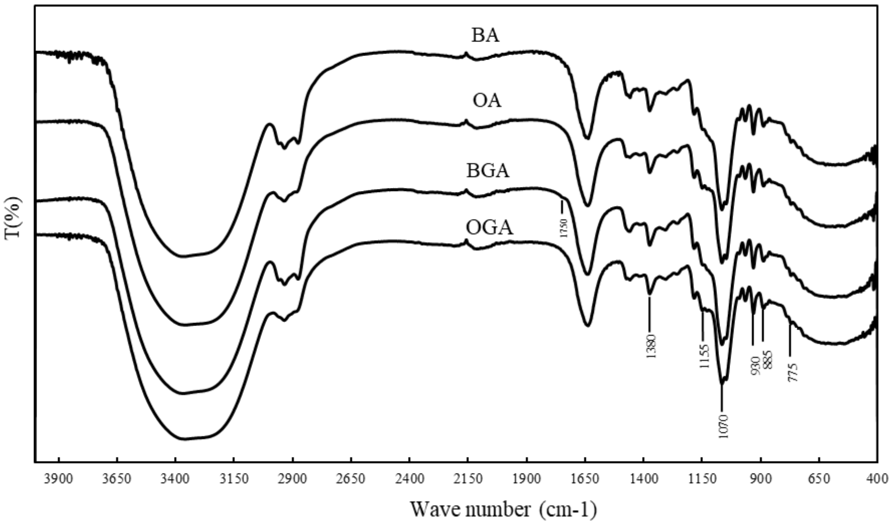

Figure S1. FTIR spectra of commercial supports butyl agarose (BA) and octyl agarose (OA) and heterofunctional supports butyl-glyoxyl agarose (BGA) and octyl-glyoxyl agarose (OGA).

51. Rueda, N.; dos Santos, J.C.S.; Torres, R.; Ortiz, C.; Barbosa, O.; Fernandez-Lafuente, R. Immobilization of Lipases on Heterofunctional Octyl–Glyoxyl Agarose Supports: Improved Stability and Prevention of the Enzyme Desorption. *Methods in Enzymology* **2016**, *571*, 73–85, doi:10.1016/BS.MIE.2015.09.035.
52. Singh, R.; Bhattacharya, B.; Tomar, S.K.; Singh, V.; Singh, P.K. Electrical, Optical and Electrophotochemical Studies on Agarose Based Biopolymer Electrolyte towards Dye Sensitized Solar Cell Application. *Measurement* **2017**, *102*, 214–219, doi:10.1016/J.MEASUREMENT.2017.02.014.
53. Trivedi, T.J.; Srivastava, D.N.R.; Rogers, R.D.; Kumar, A. Agarose Processing in Protic and Mixed Protic–Aprotic Ionic Liquids: Dissolution, Regeneration and High Conductivity, High Strength Ionogels. *Green Chemistry* **2012**, *14*, 2831–2839.
